# Non-ischemic phenotypes of low-risk chest pain patients based on exercise stress echocardiography: a pilot study

**DOI:** 10.3389/fcvm.2025.1429449

**Published:** 2025-02-19

**Authors:** Tamara Ryabova, Elena Abramenko, Ivan Yolgin, Konstantin Zavadovsky, Vyacheslav Ryabov

**Affiliations:** Cardiology Research Institute, Tomsk National Research Medical Center, Russian Academy of Sciences, Tomsk, Russia

**Keywords:** stress echocardiography, echocardiography, acute coronary syndrome, chest pain, phenotype

## Abstract

**Objective:**

A significant proportion (∼85%) of low-risk non-ST-elevation acute coronary syndrome (NSTE-ACS) patients do not receive objective confirmation of ischemia by stress echocardiography (SE), yet remain a healthcare burden due to lower long-term survival and overuse of medical services. We aimed to identify non-ischemic phenotypes in low-risk NSTE-ACS patients by analyzing a wide range of parameters available during exercise SE.

**Methods:**

Inpatients [*n* = 103, median age 56 (46–65) years, 65 (63%) men] with suspected NSTE-ACS without high-risk criteria underwent exercise SE using a semi-supine cycle ergometer. Abnormal stress biomarkers [regional wall motion abnormalities (RWMAs), ST-segment depression, induced angina, peak systolic blood pressure, force-based contractile reserve (CR), heart rate reserve (HRR), and low exercise capacity] were used for phenotyping. Non-ischemic phenotypes were identified as patients not belonging to the clusters with the highest rates of RWMA, ST-segment depression, and induced angina. Invasive or non-invasive coronary angiography was used to assess coronary anatomy.

**Results:**

The majority (90%) of patients presented with one or more abnormal stress biomarkers. Cluster analysis revealed six phenotypes, four of which were classified as non-ischemic and identified in 65 (63%) patients. Non-ischemic phenotypes differed in the prevalence of hypertensive response, reduced CR, and reduced HRR. Among patients with non-ischemic phenotypes, the incidence of coronary artery disease was low (23%).

**Conclusions:**

Four non-ischemic phenotypes of low-risk NSTE-ACS patients were identified: “near-normal type,” “inotropic insufficiency type,” “hypertensive type,” and “chronotropic insufficiency type.” Further studies are needed to investigate the long-term significance of the obtained phenotypes.

## 1 Introduction

Acute coronary syndrome (ACS) is a preliminary diagnosis that denotes recent symptoms and signs that suggest the onset of myocardial infarction (MI) or unstable angina (UA). The final diagnoses for ACS include 21% MI, 5% UA, and more than 70% attributed to other diseases and chest pain of unknown etiology (1). Approximately 500,000 ACS cases are documented in Russia annually, with approximately half of these cases classified as low-risk non-ST-elevation ACS (NSTE-ACS) (2). These patients are stable individuals with acute chest pain, not accompanied by ischemic electrocardiogram (ECG) changes and elevated levels of troponin, and they initially require verification of ischemia (3).

For this purpose, coronary computed tomography angiography (CCTA), stress imaging, or standard care with no testing are used. Among these, stress echocardiography (SE) offers several advantages: it reduces observation delays compared to standard care (4), identifies ischemia-causing coronary lesions more effectively than CCTA (5), and does not increase oncological risk. However, the main benefit is considered to be the high negative predictive value for long-term adverse cardiac events. In low-risk NSTE-ACS patients, a negative SE result, defined by the absence of regional wall motion abnormalities (RWMAs), is detected in the vast majority of cases, ranging from 65% to 97% (6, 7).

Nevertheless, in patients with unspecified non-cardiac chest pain, alongside the low incidence of cardiac events, the rate of re-presentation to the emergency department during 1-year follow-up reaches 14%, with hospital stays comparable to those of heart patients (1). Moreover, in a study by Innocenti et al., only half of the patients with cardiac events during the follow-up period were found to have positive SE results (8). Cortigiani et al. reported a significant decrease in the long-term survival of patients with reduced coronary reserve, chronotropic reserve, and a combination of both in the absence of RWMAs during dipyridamole SE (9). While other SE parameters may also be associated with a worse prognosis (10), their significance in low-risk NSTE-ACS remains uncertain.

An extended analysis of the SE results in low-risk NSTE-ACS patients may help to identify individual vulnerabilities beyond ischemia that may be associated with long-term survival and overuse of healthcare services. Also, it is essential to understand whether the parameters with proven prognostic value in stable coronary artery disease (CAD) have the potential for use in a heterogeneous population of low-risk NSTE-ACS patients and whether such “screening” is reasonable.

The aim of this study is to identify non-ischemic phenotypes in patients with low-risk NSTE-ACS by analyzing a wide range of parameters available during exercise SE using a semi-supine cycle ergometer.

## 2 Materials and methods

**Figure 1:**
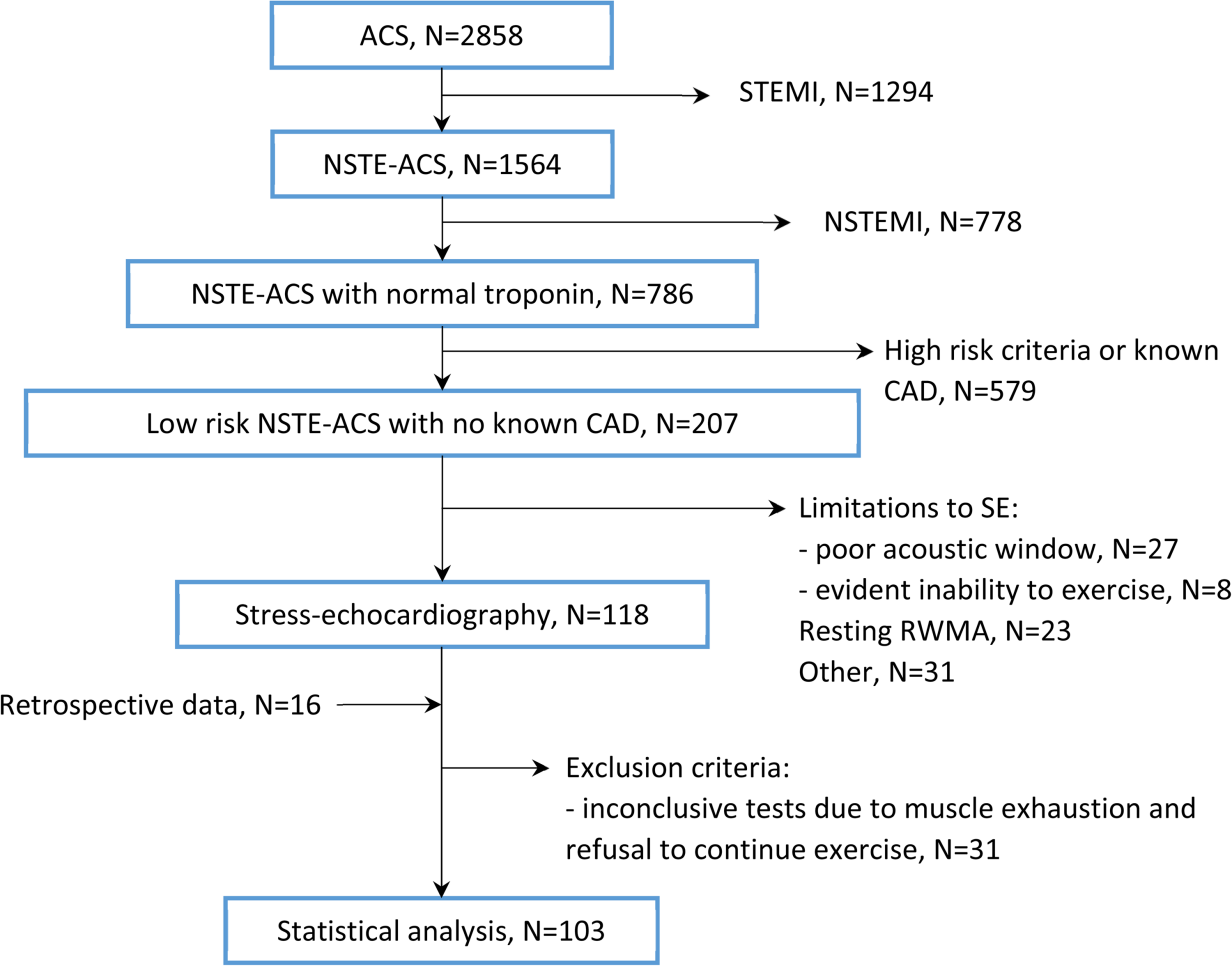
Patient flow diagram. CAD, coronary artery disease; NSTE-ACS, non-ST-elevation acute coronary syndrome; NSTEMI, non-ST-elevation myocardial infarction; RWMA, regional wall motion abnormalities; SE, stress echocardiography; STEMI, ST-elevation myocardial infarction.

The study was performed in agreement with the standards of good clinical practice and the principles outlined in the Helsinki Declaration. The study protocol was approved by the local ethics committee (protocol No. 222, dated 21 December 2021). All patients signed a written informed consent. Figure 1 shows the flowchart of patient enrollment.

### 2.1 Study population

We prospectively enrolled consecutive adult inpatients with acute chest pain of probable coronary origin, normal or non-diagnostic ECG, and negative troponin levels, who were hospitalized in the emergency cardiology department for further investigation, with a working diagnosis of low-risk NSTE-ACS in the period from January 2022 to November 2023. The troponin test was negative at least twice in a 12-h interval. Exclusion criteria included: (1) poor acoustic window; (2) resting RWMAs; (3) reduced (<50%) left ventricular ejection fraction (LVEF); (4) persistent atrial fibrillation; (5) complete atrioventricular block; (6) recent ventricular tachycardia; (7) severe valvular disease; (8) evident inability to exercise; and (9) known CAD (previous MI, revascularization, documented positive stress test, or obstructive coronary atherosclerosis). In addition, we retrospectively recruited patients who met the named criteria and underwent SE during their index hospitalization from December 2020 to December 2021.

### 2.2 Stress testing

SE was performed using a semi-supine cycle ergometer CASE (GE HealthCare, Milwaukee, WI, USA) and a Affiniti 70 Ultrasound system (Phillips, Bothell, WA, USA). The default workload protocol followed the WHO guidelines (steps of 25 W lasting 2 min, starting from 25 W). Beta-blockers or other heart rate-lowering medications were stopped 48 h before the test or were not initiated. Echocardiographic images, loop series, visual semi-quantitative contractility assessments, and blood pressure (BP) measurements were obtained at each load step and during recovery. ECG monitoring was continuously performed. Test termination criteria included: (1) stress-induced RWMAs in ≥2 adjacent myocardial segments in a 16-segment LV model; (2) ST-segment depression ≥2 mm; (3) serious arrhythmias (ventricular tachycardia, sustained ventricular allorhythmia, or hemodynamically unstable supraventricular tachycardia); (4) BP increase (systolic ≥230 mmHg, diastolic ≥120 mmHg) or decrease under load; (5) limiting chest pain or dyspnea; (6) muscle exhaustion; and (7) patient refusal to continue exercise.

End-diastolic volume, end-systolic volume (ESV), and LVEF were measured in B-mode using the Simpson method. Force-based contractile reserve (CR) was calculated as follows (11):

$$\mathrm{CR}=\frac{\mathrm{peak}\;\mathrm{systolic}\;\mathrm{BP}}{\mathrm{peak}\;\mathrm{ESV}}/\frac{\mathrm{resting}\;\mathrm{systolic}\;\mathrm{BP}}{\mathrm{resting}\;\mathrm{ESV}}$$

where BP is the blood pressure (mmHg) and ESV is the end-systolic volume (ml).

Heart rate reserve (HRR) was calculated as the ratio of peak heart rate to resting heart rate. The ST-segment shift was measured 60–80 ms after the J point. Exercise capacity (EC), expressed in metabolic equivalent (MET), was evaluated by step size and pedaling speed. Induced angina was defined as chest pain or chest discomfort with typical localization appearing during exercise.

Tests that were inconclusive due to muscle exhaustion and patient refusal to continue exercise were excluded from further analysis.

Cardiac power/mass (P/m) was calculated using the following formula (12):

$$P/m=\frac{0.222\times \mathrm{CO}\times \mathrm{mean}\;\mathrm{BP}}{\mathrm{LV}\;\mathrm{mass}}$$

where CO is the cardiac output (L/min), BP is the blood pressure (mm Hg), and LV mass is the mass of LV myocardium (g). Doppler-mode stroke volume (SV) was used to calculate cardiac output:

$$\mathrm{SV}=\mathrm{VT}I_{\mathrm{LVOT}}\;\times \;\pi \times {\left(\frac{d_{\mathrm{LVOT}}}{2}\right)}^{2}$$

where VTI_LVOT_ is the left ventricular outflow tract (LVOT) velocity time integral (VTI) and d_LVOT_ is the left ventricular outflow tract diameter.

Lung ultrasound for B-lines was carried out at rest and peak stress in four zones along the anterior chest surface, both on the left and right sides, in the projection of the apexes and anterior basal segments.

### 2.3 Phenotyping

Abnormal stress biomarkers were the basis for clustering as binary variables (presence or absence) when they occurred more often than twice. These biomarkers included: (1) RWMA in ≥2 adjacent myocardial segments; (2) ST-segment depression ≥1 mm; (3) induced angina; (4) peak systolic blood pressure (SBP) ≥220 mmHg; (5) CR <2.0; (6) HRR <1.8; and (7) low EC, defined as <6 MET for age ≤75 years, and <4 MET for age >75 years. Peak tricuspid regurgitant flow velocity (TRV) ≥3.4 m/s was not used for clustering due to missing data but was taken into account to characterize stress responses.

Non-ischemic phenotypes were defined as those not belonging to the clusters with the highest frequencies of standard ischemic criteria, such as RWMAs, ST-segment depression, and induced angina.

### 2.4 Coronary angiography

Patients underwent invasive coronary angiography or CCTA according to standard techniques during the index hospitalization. The severity of coronary atherosclerosis was evaluated retrospectively using the Gensini score (13).

### 2.5 Statistical analysis

Data were analyzed with the STATISTICA 10 program. For clustering, a hierarchical method was used (proximity measure—a percentage of disagreement, combining clusters using Ward's method). The optimal number of clusters was determined by a significant increase in the linkage distance and by an agreement that each cluster should include at least 10 patients. Comparison between >2 independent groups was conducted using the Kruskal–Wallis test for continuous variables, and the chi-square test for categorical and rank variables. Comparison between two independent groups was performed using the Mann–Whitney *U*-test for continuous variables and Fisher’s exact test for categorical variables. Correction for multiple hypothesis testing was performed using the Holm–Bonferroni method. A *p* < 0.05 was considered significant. Data are expressed as median (interquartile range) or *N* (%).

## 3 Results

### 3.1 Study design

The patient flow diagram is shown in Figure 1.

### 3.2 Patient characteristics

In total, 118 patients underwent SE during the prospective enrollment, and 16 patients were selected retrospectively. A total of 31 (23%) patients with inconclusive or uninterpretable stress tests, which were terminated due to muscle exhaustion or patient refusal to continue exercise, were excluded from further analysis. Table 1 summarizes the baseline characteristics of the 103 enrolled subjects.

**Table 1:** Baseline characteristics of the study population.

Variables	All patients (*n* = 103)
Age (years)	56 (46–65)
Sex, male (%)	65 (63%)
BMI (kg/m^2^)	28.1 (25.2–31.2)
Smoker, current or ≤5 years ago (%)	44 (42%)
Diabetes mellitus (%)	8 (8%)
Hypertension (%)	91 (89%)
Dyslipidemia (%)	75 (73%)
≥3 CAD risk factors (%)	92 (89%)
GRACE score	87 (72–109)
Carotid atherosclerosis (%)	68 (66%)

BMI, body mass index; CAD, coronary artery disease; GRACE, Global Registry of Acute Coronary Events.

### 3.3 Stress testing results

In the conventional interpretation of the stress test for ischemia, the results were positive in 14 (14%) patients, negative in 59 (57%) patients, and inconclusive in 30 (29%) patients. Reasons for early test termination were inadequate SBP increase (50%), dyspnea (33%), ST-segment depression ≥2 mm (10%), arrhythmia (7%), and induced angina (3%).

**Figure 2:**
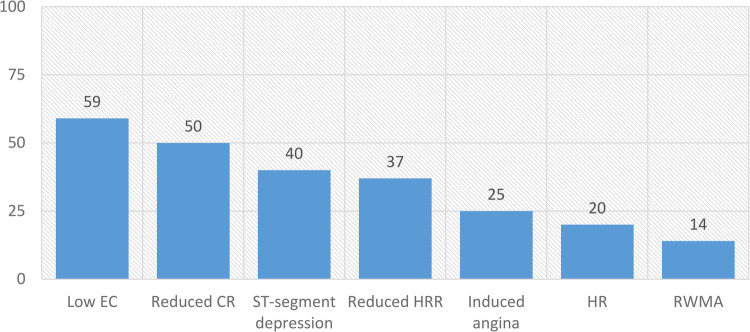
Frequencies (%) of abnormal stress biomarkers in patients with low-risk NSTE-ACS. CR, contractile reserve; EC, exercise capacity; HR, hypertensive response; HRR, heart rate reserve; RWMA, regional wall motion abnormalities.

Figure 2 shows the frequency of abnormal stress biomarkers. Individually, patients had up to six abnormal biomarkers, with only 10 (10%) patients having no abnormal biomarkers used for phenotyping.

### 3.4 Phenotyping results

**Figure 3:**
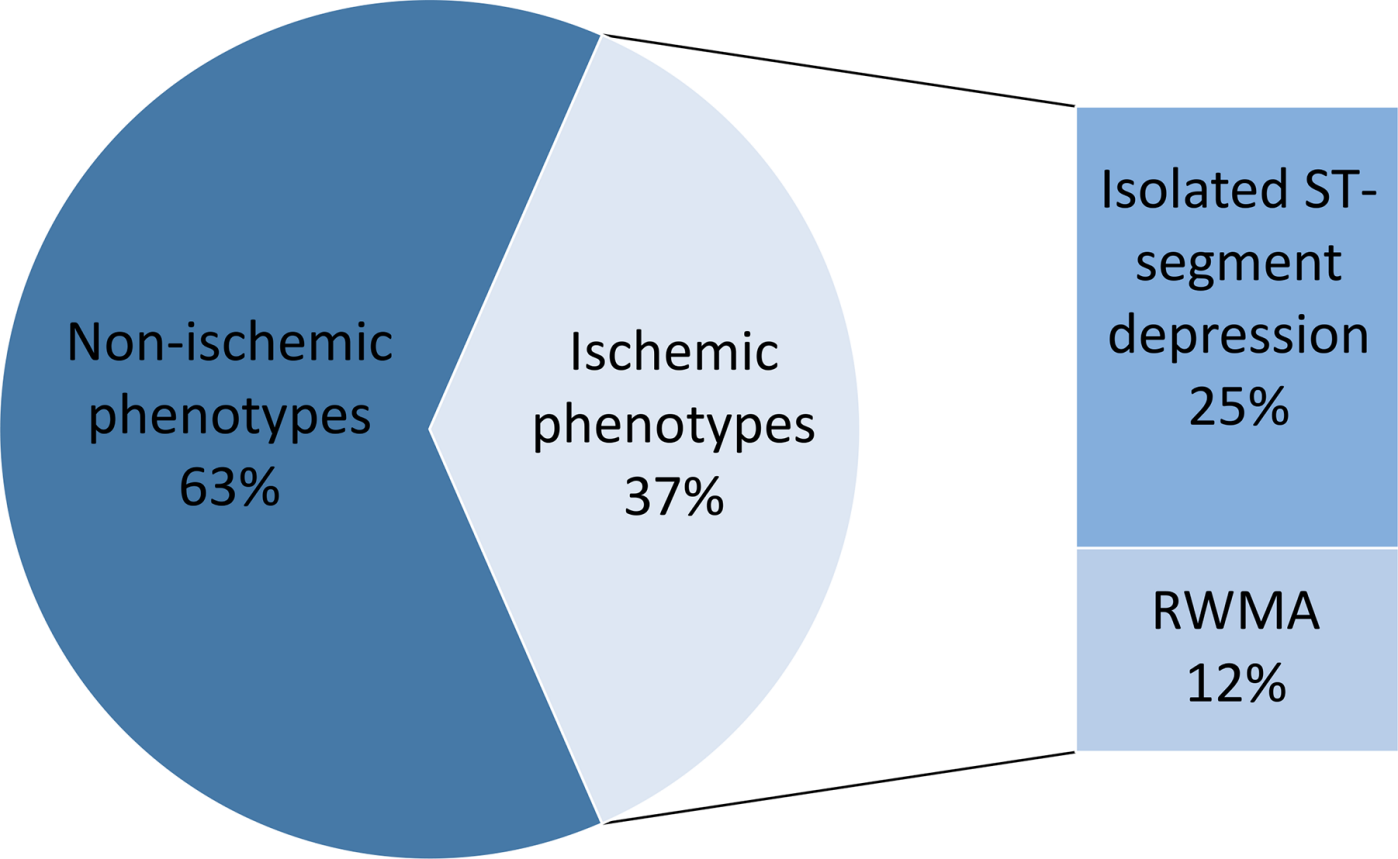
Phenotyping results. RWMA, regional wall motion abnormalities.

Cluster analysis identified six phenotypes (Table 2). The highest frequencies of ST-segment depression (100% and 83%, respectively), induced angina (35% and 83%), RWMAs (0% and 100%), and a 100% incidence of at least one of these were found in phenotypes 5 and 6. On this basis, phenotypes 5 and 6 were classified as ischemic, and the remaining phenotypes (1–4) were classified as non-ischemic (Figure 3).

**Table 2:** Frequencies of abnormal stress biomarkers in low-risk NSTE-ACS patients (phenotyping results).

Type No.	*N*	Low exercise capacity (%)	Hypertensive response (%)	ST-segment depression (%)	Induced angina (%)	RWMA (%)	Reduced contractile reserve (%)	Reduced heart rate reserve (%)
Non-ischemic phenotypes								
1	17	41	0	0	0	6	0	0
2	10	40	30	0	0	0	100	0
3	10	40	100	20	10	0	0	0
4	28	79	14	11	21	4	64	100
Ischemic phenotypes								
5	26	62	12	100	35	0	46	23
6	12	67	8	83	83	100	92	33

RWMAs, regional wall motion abnormalities.

Non-ischemic types differed in the frequency of hypertensive response (HR), reduced CR, and reduced HRR. The rate of low exercise capacity varied from 40% to 79% and did not show significant differences between the phenotypes. The frequency rate of standard ischemic criteria did not exceed the first quartile. Two patients who had inducible RWMAs showed an abnormality pattern related to non-ischemic phenotypes and were categorized into phenotypes 1 and 4. One of them had no atherosclerotic plaques (false-positive stress test result), and the other had borderline stenoses (50%–60%) in two arteries, with fractional flow reserve not being assessed.

### 3.5 Non-ischemic phenotypes

Phenotype 1 patients (*n* = 17) had a minimal incidence of abnormalities (“near-normal type”); these patients typically exhibited solely lowered exercise capacity or no abnormal stress biomarkers. One patient had inducible RWMAs unaccompanied by other signs of ischemia (Table 2).

All patients classified as phenotype 2 (*n* = 10) had reduced force-based contractile reserve without other signs of ischemia (“inotropic insufficiency type”). They showed a smaller absolute increase in peak LVEF, 5 (3–7) vs. 12 (8–15) in patients with normal CR (types 1 and 3, *p* < 0.001), higher LVEF at rest, 66 (63–71) vs. 58 (57–61), *p* < 0.001, smaller ESV (*p* = 0.017), and a smaller decrease in ESV at peak load, 4 (2–5) ml vs. 12 (9–18) ml, *p* < 0.001. Peak SV and its peak increase in these groups did not differ (*p* = 0.984 and 0.564) (Table 3).

**Table 3:** Clinical and instrumental characteristics for non-ischemic phenotypes of low-risk NSTE-ACS patients.

	All patients	Type 1	Type 2	Type 3	Type 4	*р*	
*N*	65	17 (27%)	10 (15%)	10 (15%)	28 (43%)	NA	
Abnormal stress biomarkers^a^	2 (1–3)	0 (0–1)^2,3,4^	2 (1–2)^1,4^	2 (1–2)^1,4^	3 (2–4)^1,2,3^	< 0.001	
Abnormal stress biomarkers defining the type							
Contractile reserve^a^	2.1 (1.8–2.5)	2.3 (2.1–2.7)^2,4^	1.8 (1.7–1.9)^1,3^	2.6 (2.3–3.0)^2,4^	1.9 (1.5–2.2)^1,3^	< 0.001	
Heart rate reserve^a^	1.9 (1.6–2.1)	2.0 (1.9–2.3)^4^	2.1 (2.0–2.5)^4^	2.1 (2.0–2.2)^4^	1.6 (1.5–1.7)^1,2,3^	< 0.001	
Peak SBP (mmHg)^a^	199 (180–222)	191 (187–204)^3^	197 (178–222)^3^	238 (230–246)^1,2,4^	187 (165–208)^3^	0.015	
Abnormal stress biomarkers not used for classification							
В-lines, *n* (%)^b^	8 (14%)	1 (6%)	1 (14%)	2 (20%)	4 (17%)	0.717	
TRV >3.4 сm/s (%)^c^	12 (31%)	2 (22%)	3 (42%)	2 (33%)	5 (38%)	0.702	
Е/e′ > 14 (%)	1 (2%)	0	0	1 (10%)	0	0.134	
Left ventricular contractility							
ΔLVEF (%)^a^	9 (5–13)	12 (10–16)^2^	5 (3–7)^1,3^	11 (7–13)^2^	9 (3–12)	0.004	
ΔSV (%)	16 (7–26)	15 (7–20)	19 (10–30)	15 (10–19)	17 (5–26)	0.871	
Exercise capacity							
MET^a^	5.7 (4.6–6.4)	6.1 (5.6–7.2)^4^	5.5 (5.3–6.4)	6.0 (5.2–6.8)	4.7 (4.0–5.9)^1^	0.021	
Inconclusive test (%)	25 (38%)	1 (6%)^3,4^	2 (20%)^3^	9 (90%)^1,2^	13 (46%)^1^	<0.001	
Cardiac power/mass							
P/m at rest (W/100 g)^a^	0.7 (0.6–0.9)	0.6 (0.6–0.7)^3,4^	0.6 (0.5–0.7)	0.5 (0.4–0.6)^1,4^	0.9 (0.6–1.1)^1,3^	<0.001	
Peak P/m (W/100 g)	2.1 (1.7–2.4)	2.1 (1.6–2.5)	2.2 (2.0–2.5)	1.9 (1.8–2.0)	2.1 (1.5–2.4)	0.506	
P/m reserve (W/100 g)	1.3 (1.0–1.8)	1.4 (1.1–1.8)	1.7 (1.4–1.8)	1.3 (1.3–1.6)	1.2 (0.7–1.5)	0.187	
Clinical profile of the patients							
Age (years)	55 (45–65)	54 (47–62)	53 (42–67)	53 (42–62)	56 (47–67)	0.672	
Male subjects (%)	43 (66%)	13 (76%)	7 (70%)	8 (80%)	15 (54%)	0.295	
BMI (kg/m^2^)	28.6 (25.4–31.7)	27.4 (24.2–29.3)	27.0 (23.8–30.7)	30.4 (28.1–32.5)	28.9 (26.2–32.8)	0.110	
Smoker, current or ≤5 years ago (%)	29 (45%)	8 (47%)	6 (60%)	5 (50%)	10 (36%)	0.569	
Dyslipidemia (%)	46 (71%)	13 (76%)	6 (60%)	7 (70%)	20 (71%)	0.841	
Diabetes mellitus (%)	6 (9%)	1 (6%)	1 (10%)	0	4 (14%)	0.551	
Hypertension (%)	57 (89%)	14 (82%)	9 (90%)	10 (100%)	25 (89%)	0.562	
Chronic pulmonary disease (COPD, asthma) (%)	2 (3%)	0	0	0	2 (7%)	0.436	
Chronic renal disease, GFR < 60 ml/kg/min (%)	5 (8%)	0	1 (10%)	0	4 (14%)	0.257	
Resting and peak cardiac parameters							
Resting heart rate (bpm)^a^	68 (60–82)	67 (59–72)^4^	64 (59–67)^4^	58 (55–63)^4^	83 (73–87)^1,2,3^	< 0.001	
Peak heart rate^a^	136 (122–146)	141 (131–148)	142 (126–153)	127 (117–137)	130 (113–145)	0.029	
Resting blood pressure (mmHg)							
Systolic	128 (119–139)	124 (115–131)	134 (114–140)	130 (122–138)	131 (120–144)	0.577	
Diastolic	79 (72–88)	73 (67–79)	82 (67–89)	86 (77–91)	80 (73–87)	0.167	
Peak blood pressure (mmHg)							
Systolic^a^	199 (180–222)	191 (187–204)^3^	197 (178–222)^3^	238 (230–246)^1,2,4^	187 (165–208)^3^	< 0.001	
Diastolic^a^	89 (82–104)	86 (80–92)^3^	99 (81–119)	108 (100–111)^1,4^	87 (79–98)^3^	0.015	
Resting EDV (ml)	98 (82–110)	96 (90–110)	98 (91–105)	108 (98–122)	92 (76–105)	0.095	
Peak EDV (ml)	93 (80–110)	92 (82–102)	107 (80–116)	105 (91–115)	92 (80–105)	0.467	
Resting end-diastolic index (ml/m^2^)	50 (44–54)	51 (43–57)	51 (47–55)	51 (49–57)	46 (43–52)	0.181	
Peak end-diastolic index (ml/m^2^)	48 (43–54)	46 (42–53)	57 (43–64)	50 (45–57)	47 (44–51)	0.263	
Resting ESV (ml)^a^	36 (28–44)	39 (32–41)	30 (25–40)^3^	47 (43–50)^2,4^	34 (28–42)^3^	0.005	
Peak ESV (ml)	28 (20–32)	19 (16–24)	24 (21–38)	33 (32–37)	25 (20–30)	0.061	
Resting end-systolic index (ml/m^2^)^a^	19 (15–23)	19 (16–24)	16 (13–20)	22 (20–25)^4^	16 (14–20)^3^	0.015	
Peak end-systolic index (ml/m^2^)	13 (11–16)	13 (10–15)	13 (11–19)	16 (15–17)	13 (11–16)	0.174	
Resting SV (ml)	64 (56–69)	63 (54–69)	64 (53–73)	65 (61–69)	62 (56–68)	0.826	
Peak SV (ml)	72 (64–79)	70 (65–80)	75 (61–79)	74 (72–84)	68 (64–79)	0.736	
Resting stroke index (ml/m^2^)	31 (29–36)	31 (29–36)	33 (30–37)	31 (29–34)	31 (28–36)	0.871	
Peak stroke index (ml/m^2^)	37 (33–41)	36 (34–39)	37 (37–45)	36 (33–40)	37 (33–42)	0.727	
Resting LV EF (%)^a^	61 (58–67)	60 (58–61)^2^	66 (63–71)^1,3^	57 (55–58)^2,4^	63 (59–67)^3^	0.001	
Peak LV EF (%)	71 (68–75)	71 (71–76)	72 (68–76)	69 (65–71)	72 (68–75)	0.066	
Resting cardiac output (L/min)^a^	2.2 (1.9–2.6)	2.1 (1.9–2.3)^4^	2.1 (1.9–3.7)	1.8 (1.7–2.0)^4^	2.5 (2.2–3.4)^1,3^	0.002	
Peak cardiac output (L/min)	5.1 (4.4–6.3)	4.8 (4.6–5.7)	6.2 (4.4–11.2)	4.5 (4.1–5.4)	5.3 (4.2–7.5)	0.445	
Resting cardiac index (L/min/m^2^)^a^	1.2 (0.9–1.5)	1.2 (0.9–1.2)	1.1 (1.1–2.2)	0.9 (0.8–1.0)^4^	1.5 (1.1–1.9)^3^	0.004	
Peak cardiac index (L/min/m^2^)	2.6 (2.2–3.3)	2.6 (2.4–3.0)	3.1 (2.6–5.9)	2.2 (1.8–2.6)	3.1 (2.1–3.9)	0.128	
Resting Е/е′	6.4 (5.4–7.5)	6.3 (5.7–6.9)	6.3 (4.7–6.8)	7.4 (6.2–12.0)	6.0 (5.3–8.3)	0.203	
Peak Е/е′^a^	6.9 (5.7–7.9)	6.2 (5.8–6.9)	5.3 (4.2–6.7)	7.3 (6.1–8.6)	7.6 (5.7–9.3)	0.025	
LV mass (g)^a^	164 (143–194)	148 (143–164)^3^	176 (158–192)^3^	206 (188–241)^1,2,4^	158 (133–196)^3^	0.006	
LV mass index (g/m^2^)^a^	86 (75–98)	81 (73–86)^3^	90 (79–101)	97 (89–122)^1,4^	83 (71–95)^3^	0.010	
Left atria index (ml/m^2^)	28 (26–33)	27 (26–32)	29 (26–33)	30 (29–36)	27 (26–30)	0.091	
Coronary atherosclerosis							
Coronary angiography (%)	55 (85%)	14 (82%)	6 (60%)	10 (100%)	25 (89%)	NA	
ICA (*n*)/CCTA (*n*)	9/46	1/13	1/5	1/9	6/19	NA	
Gensini score 5	5.0 (0–11.5)	5.5 (0–15.0)	5.0 (0–6.0)	5.3 (3.5–13.0)	5.0 (0–8.5)	0.739	
CAD, ≥50% lesions (%)	15 (23%)	5 (36%)	2 (33%)	3 (30%)	5 (20%)	0.768	
CAD, ≥70% lesions (%)	6 (9%)	1 (7%)	1 (17%)	2 (20%)	2 (8%)	0.632	

BMI, body mass index; CAD, coronary artery disease; CCTA, coronary computed tomography angiography; COPD, chronic obstructive pulmonary disease; EDV, end-diastolic volume; EF, ejection fraction; ESV, end-systolic volume; GFR, glomerular filtration rate; ICA, invasive coronary angiography; LV, left ventricle; MET, metabolic equivalent; NA, not applicable; P/m, power/mass; SBP, systolic blood pressure; SV, stroke volume; TRV, tricuspid regurgitant flow velocity.

^a^ Significant differences: the superscript indicates the number of the group against which the difference was observed.

^b^ Missing data for 1, 3, and 6 patients of 1, 2, and 4 phenotypes, respectively.

^c^ 8, 3, 4, and 15 patients of 1, 2, 3, and 4 phenotypes, respectively, were not available for the TRV assessment.

Phenotype 3 patients were characterized by a hypertensive response to exercise (“hypertensive type”). In nine (90%) of them, the stress test was completed before reaching the submaximal heart rate due to a rapid growth of SBP. Despite this, they had a normal HRR and a lower resting heart rate than other phenotypes. Of all the subjects studied, these patients had the highest LV myocardial mass index. There were eight (80%) men in this phenotype group, but the sex differences between the groups were insignificant.

Patients classified as phenotype 4 (“chronotropic insufficiency type”) had the highest incidence of abnormal stress biomarkers. They had reduced HRR, accompanied by reduced CR (64%) and low exercise capacity (79%). This group was characterized by the highest resting heart rate, 83 (73–87) bpm vs. 63 (58–70) bpm in the other groups (*p* < 0.001), and the highest associated indices of pumping function (cardiac output, cardiac index, and P/m). It is worth noting that the target heart rate was not achieved in only 46% of these patients. A subanalysis of phenotype 4 patients who achieved (*n* = 16) or did not achieve (*n* = 12) submaximal heart rate showed significant differences in resting heart rate, 86 (83–90) bpm vs. 72 (65–81) bpm, *p* = 0.001, peak heart rate, 144 (130–148) bpm vs. 112 (98–120) bpm, *p* < 0.001, the ratio of achieved to submaximal heart rate, 85% (85%–86%) vs. 64% (61%–75%), *p* < 0.001, and heart rate reserve, 1.7 (1.6–1.7) vs. 1.5 (1.3–1.6), *p* = 0.022. Inotropic insufficiency was detected in these subgroups with the same frequency, *p* = 1.000; exercise capacity tended to decrease in patients who stopped the test prematurely, 4.2 (3.8–5.7) MET vs. 5.2 (4.4–6.4) MET, *p* = 0.170.

Baseline clinical and demographic characteristics did not differ by phenotype, except for higher body mass index (BMI) in “hypertensive type” patients. Detailed characteristics of the phenotypes are presented in Table 3.

### 3.6 Cardiac power/mass

Resting P/m was higher in the “chronotropic insufficiency type” patients, essentially due to higher heart rate at rest, since no other inputs for P/m calculation showed significant differences between this group and the others (blood pressure, stroke volume, and LV mass). Peak P/m and P/m reserve did not differ between phenotypes. A subanalysis of all non-ischemic phenotype patients with low (*n* = 37) and normal (*n* = 28) exercise capacity, irrespective of phenotype, revealed a lower P/m reserve in patients with low exercise capacity, 1.3 (0.8–1.5) vs. 1.6 (1.2–1.8), *p* = 0.012.

### 3.7 Coronary atherosclerosis

In total, 92 (89%) patients underwent coronary angiography (29 invasive coronary angiography, 63 CCTA). Obstructive atherosclerosis with ≥50% narrowing in any coronary artery or branch was identified in 31 (34%) patients; with ≥70% narrowing in 17 (18%) patients. Multivessel disease (≥2) was found in 16 (17%) patients. Twenty-eight (30%) patients had non-obstructive coronary lesions, and 33 (36%) had no atherosclerotic plaques. The Gensini score was 5.0 (0–14.8) in all patients, 13.3 (8.0–17.5) in patients with 50%–69% stenosis, and 30.0 (24.5–46.0) in patients with ≥70% stenosis.

There were no differences between the non-ischemic phenotypes in terms of the Gensini score (*p* = 0.734) and the frequency of CAD (Table 3). Lesions ≥70%, detected in 6 (9%) patients, did not impair local contractility. Compared with ischemic phenotypes, non-ischemic phenotype patients had a lower incidence of CAD, both ≥50% and ≥70% lesions (*p* = 0.048 and 0.013, respectively).

## 4 Discussion

Our hospital works as a regional PCI center (14). Local practice does not include prehospital non-invasive testing of patients presenting with chest pain; the decision on whether hospitalization is necessary is based on an initial ACS assessment (3). Thus, all enrolled patients with acute chest pain, normal or non-diagnostic ECG, and normal troponin levels had a high clinical expectation of CAD according to clinical judgment based on history and pain characteristics. Compared to the other studies with the same design (4, 15), our patients were similar in age and sex but had a higher frequency of traditional CAD risk factors, mainly dyslipidemia and hypertension, and the majority of them (89%) had ≥3 risk factors.

However, the incidence of inducible RWMA (14%) in the study group was low, although higher than that reported by Jasani et al. (5%) and Heitner et al. (8%) (4, 15, 16). In addition, 30% of patients had isolated ST-segment depression, and 7% experienced induced angina not associated with RWMA or ST-segment shift, two of the obligatory criteria for microvascular ischemia (17).

The most frequent abnormal stress biomarker not directly related to ischemia was low exercise capacity. The chosen MET threshold was quite low and was considered, along with the failure to achieve submaximal heart rate, to be a criterion for an inconclusive stress test (18). The incidence of low exercise capacity in ischemic and non-ischemic types, 63% and 57%, did not differ. Its largest proportion (79%) was found in “chronotropic insufficiency type” patients. As low exercise capacity was detected in the absence of other abnormalities and was not associated with failure to achieve submaximal heart rate, it may be the consequence of physical detraining. It may also be the result of performing a stress load on a semi-supine bicycle ergometer. According to Flores-Blanc et al., low exercise capacity at the same METs threshold was observed in only 8% of chest pain patients (the sample included individuals with known CAD) who performed the exercise on a treadmill (19).

Inotropic insufficiency, which is a component of the ischemic response, was determined in half of the patients. It reflects the inability of the ischemic myocardium to provide the necessary increase in SBP, and the absence of hyperkinesia does not produce the required reduction in ESV. In patients with stable CAD, a high percentage of reduced CR is accompanied by a comparable frequency of RWMA and reduced coronary reserve (11). In our study, this response was also detected in combination with RWMA. However, occurring in isolation, reduced CR is more likely to be of “artifact” origin. It is observed in patients with a higher resting LVEF who have a low reserve for reducing ESV. In addition, given the absence of other traditional ischemia criteria and the low frequency of obstructive lesions, it can be judged that it is not a reflection of balanced ischemia. There is also little reason to suspect that this is an early sign of diastolic dysfunction, as this group revealed the lowest peak Е/е′ with a tendency to decrease compared to the resting value and the low frequency of appearance or increase in the number of B-lines.

An increase in heart rate of less than 80% of the resting value was detected in 37% of patients. When associated with signs of ischemia, this could indicate a low ischemic threshold; however, this response was also observed in the pattern of non-ischemic responses. Cluster analysis identified a group of patients with a 100% incidence of reduced chronotropic reserve not associated with ischemia, combining into it those who reached a submaximal heart rate but had a higher resting heart rate and those who prematurely stopped the exercise, more often due to limiting dyspnea or a high rate of systolic BP increase, in other words, having an increased sympathetic tone or other conditions requiring correction. This emphasizes the heterogeneity of the population of patients with low-risk NSTE-ACS in the initial cardiac function parameters. Differences in response to exercise also complicate the picture. Pharmacological stress agents, mostly vasodilators, are more standardized because the response is assessed after the administration of a standard dose of the drug. The study by Cortigiani et al. showed the prognostic value of a reduced chronotropic reserve in acute chest pain patients and found a reduced 8-year survival rate of 76% vs. 93%; this study utilized dipyridamole, and some tests were carried out under the influence of beta-blockers; also, the study sample included patients with atrial fibrillation. It is worth noting that the age of patients with a reduced chronotropic reserve in that study was higher, 71 ± 12 years (increasing to 75 ± 11 years in combination with a reduction in coronary reserve) (9).

Every fifth patient exhibited a hypertensive response. Cluster analysis confirmed that this response mainly interferes with obtaining diagnostic results from exercise tests. In 90% of the “hypertensive type” patients, SE was incomplete. Higher LV mass and LV mass index may indicate the duration of hypertension, which is one of the factors causing microvascular remodeling and the development of microvascular ischemia (17). At the same time, ST-segment depression and induced angina were observed at a low rate in these patients, although it is possible that the ischemic threshold was not reached in others.

One-sixth of the patients experienced stress-induced pulmonary hypertension. In the absence of ischemia, this response is considered a marker of the initial stage of pulmonary arterial hypertension in asymptomatic patients with risk factors (20). In a significant proportion of the patients, the flow of tricuspid regurgitation could not be visualized (in 41% at rest and 45% at peak load), which is more typical of healthy individuals (21). Despite this assumption, the response was not used for clustering, and in non-ischemic phenotypes, isolated increases in systolic pulmonary artery pressure were detected with a similar frequency.

Rare abnormalities were a positive diastolic stress test (1%) and an arrhythmia meeting stress termination criteria (2%).

We used the cardiac power indices to approximate the prognosis of patients with different phenotypes. Their prognostic value was shown in a large study by Anand et al., which assessed the outcomes of a long-term, 3.9 (0.6–8.3) years, follow-up of 24,885 stable patients with preserved ejection fraction (≥50%) without valvular heart disease who performed routine exercise stress echocardiography on a treadmill (99%) or a bicycle ergometer (1%). Patients with lower peak P/m and P/m reserve had lower survival rates and a higher incidence of heart failure (12). In our study, non-ischemic phenotypes did not differ in terms of peak P/m and P/m reserve.

Worth considering is that in patients with non-high-risk NSTE-ACS, particularly those with no known CAD, and in stable patients who were included in Anand et al.’s study, the overall mortality during the observation period was quite low (8, 12). Also, in a series of studies devoted to the role of stress echocardiography in the diagnostic algorithm for acute chest pain, the study by Cortigiani et al. stood out, characterized by the highest level of overall patient mortality (24%) and the longest follow-up period (7.3 ± 4.3 years) (9).

Pooling patients into homogeneous groups (phenotypes) within the same disease and applying a differentiated approach to their treatment is based on differences in long-term prognosis, more often adverse cardiac events. A well-known example of phenotyping using stress echocardiographic parameters is the StressEcho 2020 study. Its authors supplemented the assessment of local contractility with contractile reserve, coronary reserve, heart rate reserve, and lung ultrasound for B-lines, identifying the phenotypes of patients with stable CAD (ischemic, congestive, impaired contractility, microvascular, and autonomic dysfunction) and showed that long-term survival decreases with increasing number of abnormalities (11). However, the task of functional phenotyping in low-risk acute chest pain patients without evidence of ischemia due to the lack of a common diagnosis is closer to determining indicators for screening in the general population.

Now, in the era of highly sensitive troponin tests, there is a growing question about the utility of non-invasive tests, particularly stress tests, in suspected ACS, supported by data on a good mid-term prognosis for survival without cardiovascular events (22). In certain categories of patients, stress tests may prolong the path to diagnosis and resource utilization, for example in women with a narrow anteroposterior chest dimension and non-high CAD probability, due to the increased likelihood of having a false-positive SE, which may be the result of external compression rather than true myocardial ischemia (23). Summarizing the current evidence, it can be assumed that in view of the expanded diagnostic capabilities of exercise SE beyond the assessment of local contractility, the uncertainty surrounding the longer-term prognosis of patients with impaired cardiac reserve makes the question of the reasonable “screening” with SE still relevant. Therefore, larger studies with more participants and longer follow-up durations are needed to assess the survival of these patients. In addition, evaluating other, equally important endpoints with a higher incidence, such as the rate of emergency department re-presentations, makes practical sense closer to real time. The results of such an evaluation have not yet been reported.

### 4.1 Limitations

Clinical risk stratification tools, such as the HEART score, which could improve the objectivity of triage, were not applied (as they are not routinely used in local practice). A distinctive feature of the patient sample, unlike other similar studies, is the higher incidence of hypertension among CAD risk factors, which makes us cautious about these data. It was assumed that the results of invasive and non-invasive coronary angiography for anatomic assessment of the coronary arteries are considered equal. The rather small size of some clusters may affect the representability of the findings. Pulmonary congestion was not evaluated definitively by B-line criteria because of missing data.

## 5 Conclusion

The predominant majority (90%) of low-risk NSTE-ACS patients have one or more abnormal stress biomarkers based on an extended stress test assessment. Four non-ischemic phenotypes were identified: “near-normal type,” “inotropic insufficiency type,” “hypertensive type,” and “chronotropic insufficiency type.” Non-ischemic phenotypes exhibit a low frequency of obstructive coronary atherosclerosis. Further proof-of-concept studies are needed to investigate the significance of the obtained phenotypes in the long term.

## Data Availability

The original contributions presented in the study are included in the article/Supplementary Material, further inquiries can be directed to the corresponding author.

## References

[1] Mol KA Smoczynska A Rahel BM Meeder JG Janssen L Doevendans PA Non-cardiac chest pain: prognosis and secondary healthcare utilisation. Open Heart. (2018) 5(2):e000859. 10.1136/openhrt-2018-000859 30364505 PMC6196943

[2] Alekyan BG Boytsov SA Manoshkina EM Ganyukov VI. Myocardial revascularization in Russian Federation for acute coronary syndrome in 2016–2020. Kardiologiia. (2021) 61(12):4–15. 10.18087/cardio.2021.12.n1879 35057716

[3] Byrne RA Rossello X Coughlan JJ Barbato E Berry C Chieffo A 2023 ESC guidelines for the management of acute coronary syndromes. Eur Heart J. (2023) 44(38):3720–826. 10.1093/eurheartj/ehad191 37622654

[4] Jasani G Papas M Patel AJ Jasani N Levine B Zhang Y Immediate stress echocardiography for low-risk chest pain patients in the emergency department: a prospective observational cohort study. J Emerg Med. (2018) 54(2):156–64. 10.1016/j.jemermed.2017.10.019 29274930

[5] Danad I Szymonifka J Twisk JWR Norgaard BL Zarins CK Knaapen P Diagnostic performance of cardiac imaging methods to diagnose ischaemia-causing coronary artery disease when directly compared with fractional flow reserve as a reference standard: a meta-analysis. Eur Heart J. (2017) 38(13):991–8. 10.1093/eurheartj/ehw095 27141095 PMC5381594

[6] Langdorf MI Wei E Ghobadi A Rudkin SE Lotfipour S. Echocardiography to supplement stress electrocardiography in emergency department chest pain patients. West J Emerg Med. (2010) 11(4):379–83. 21079713 PMC2967693

[7] Piñeiro-Portela M Peteiro-Vázquez J Bouzas-Mosquera A Martínez-Ruiz D Yañez-Wonenburger JC Pombo F Comparison of two strategies in a chest pain unit: stress echocardiography and multidetector computed tomography. Rev Esp Cardiol (Engl Ed). (2021) 74(1):59–64 (in English, Spanish). 10.1016/j.recesp.2020.01.007 32402688

[8] Innocenti F Cerabona P Donnini C Conti A Zanobetti M Pini R. Long-term prognostic value of stress echocardiography in patients presenting to the ED with spontaneous chest pain. Am J Emerg Med. (2014) 32(7):731–6. 10.1016/j.ajem.2014.03.026 24768667

[9] Cortigiani L Vecchi A Bovenzi F Picano E. Reduced coronary flow velocity reserve and blunted heart rate reserve identify a higher risk group in patients with chest pain and negative emergency department evaluation. Intern Emerg Med. (2022) 17(7):2103–11. 10.1007/s11739-022-03018-5 35864372

[10] Pellikka PA Arruda-Olson A Chaudhry FA Chen MH Marshall JE Porter TR Guidelines for performance, interpretation, and application of stress echocardiography in ischemic heart disease: from the American Society of Echocardiography. J Am Soc Echocardiogr. (2020) 33(1):1–41.e8. 10.1016/j.echo.2019.07.001 31740370

[11] Ciampi Q Zagatina A Cortigiani L Wierzbowska-Drabik K Kasprzak JD Haberka M Prognostic value of stress echocardiography assessed by the ABCDE protocol. Eur Heart J. (2021) 42(37):3869–78. 10.1093/eurheartj/ehab493 34449837 PMC8486488

[12] Anand V Kane GC Scott CG Pislaru SV Adigun RO McCully RB Prognostic value of peak stress cardiac power in patients with normal ejection fraction undergoing exercise stress echocardiography. Eur Heart J. (2021) 42(7):776–85. 10.1093/eurheartj/ehaa941 33377479

[13] Rampidis GP Benetos G Benz DC Giannopoulos AA Buechel RR. A guide for Gensini score calculation. Atherosclerosis. (2019) 287:181–3. 10.1016/j.atherosclerosis.2019.05.012 31104809

[14] Ryabov VV Gombozhapova AE Demyanov SV. Profile of a patient with non-ST segment elevation myocardial infarction in actual clinical practice. Russian J Cardiol. (2021) 26(2):4071. 10.15829/1560-4071-2021-4071

[15] Heitner JF Klem I Rasheed D Chandra A Kim HW Van Assche LM Stress cardiac MR imaging compared with stress echocardiography in the early evaluation of patients who present to the emergency department with intermediate-risk chest pain. Radiology. (2014) 271(1):56–64. 10.1148/radiol.13130557 24475814 PMC4263624

[16] Abramenko EE Ryabova TR Yolgin II Ryabov VV. Diagnostic value of exercise stress echocardiography on a horizontal cycle ergometer in patients with low-risk non-ST elevation acute coronary syndrome. Russian J Cardiol. (2023) 28(8):5409 (in Russian). 10.15829/1560-4071-2023-5409

[17] Ong P Camici PG Beltrame JF Crea F Shimokawa H Sechtem U International standardization of diagnostic criteria for microvascular angina. Int J Cardiol. (2018) 250:16–20. 10.1016/j.ijcard.2017.08.068 29031990

[18] Stein RA Chaitman BR Balady GJ Fleg JL Limacher MC Pina IL Safety and utility of exercise testing in emergency room chest pain centers: an advisory from the Committee on Exercise, Rehabilitation, and Prevention, Council on Clinical Cardiology, American Heart Association. Circulation. (2000 ) 102(12):1463–7. 10.1161/01.CIR.102.12.1463 10993869

[19] Flores-Blanco PJ Cambronero F García-Navarro M de la Morena G Valdés M Manzano-Fernández S. Inconclusive exercise stress echocardiography in patients with chest pain: prevalence and clinical determinants. Rev Esp Cardiol (Engl Ed). (2018) 71(5):406–8 (in English, Spanish). 10.1016/j.recesp.2017.02.020 28499844

[20] Lopes LR Loureiro MJ Miranda R Almeida S Almeida AR Cordeiro A The usefulness of contrast during exercise echocardiography for the assessment of systolic pulmonary pressure. Cardiovasc Ultrasound. (2008) 6:51. 10.1186/1476-7120-6-51 18851729 PMC2570360

[21] Wierzbowska-Drabik K Picano E Bossone E Ciampi Q Lipiec P Kasprzak JD. The feasibility and clinical implication of tricuspid regurgitant velocity and pulmonary flow acceleration time evaluation for pulmonary pressure assessment during exercise stress echocardiography. Eur Heart J Cardiovasc Imaging. (2019) 20(9):1027–34. 10.1093/ehjci/jez029 30824900

[22] Mark DG Huang J Ballard DW Vinson DR Rana JS Sax DR Emergency department referral of patients with chest pain for noninvasive cardiac testing and 2-year clinical outcomes. Circ Cardiovasc Qual Outcomes. (2024) 17(6):e010457. 10.1161/CIRCOUTCOMES.123.010457 38779848

[23] Sonaglioni A Rigamonti E Nicolosi GL Lombardo M. Appropriate use criteria implementation with modified Haller index for predicting stress echocardiographic results and outcome in a population of patients with suspected coronary artery disease. Int J Cardiovasc Imaging. (2021) 37(10):2917–30. 10.1007/s10554-021-02274-4 33961159

